# An Audit to Assess the Level Pregnancy Screening Conducted on Admission for Female Inpatients on an Acute Psychiatric Ward

**DOI:** 10.1192/bjo.2022.440

**Published:** 2022-06-20

**Authors:** Nikhita Handa, Jessica Quinlan, Mariam Mohammed

**Affiliations:** 1 Greater Manchester Mental Health Trust, Manchester, United Kingdom; 2 Wrightington, Wigan and Leigh Teaching Hospitals NHS Foundation Trust, Wigan, United Kingdom

## Abstract

**Aims:**

Currently, practice is that if patients of childbearing age provide a urine sample on admission they will also be consented to test for pregnancy. As many new patients may refuse to provide a urine sample often due to their mental state or concerns about drug testing this results in some patients not being tested for pregnancy during admission unless required for medication or at patient request. Given the high level of vulnerability and the medication implications for pregnant patients, ascertaining pregnancy status early on in admission is beneficial to patients found to be pregnant. Therefore, we aimed to audit how pregnancy status is assessed and documented on admission and aim to improve the practice where areas for development are identified.

**Methods:**

Over the 6 month period July-December 2021 there were 105 inpatient admissions on an acute female psychiatry ward. Using a random number generator 15 patients from this cohort were selected and their notes audited as to whether a urine pregnancy test or bHCG serum pregnancy test was completed on admission. If not, we searched the admission notes for documentation of ‘pregnancy, last menstrual period (LMP), sexually active status, contraceptive use’.

**Results:**

Of the 15 patients audited, 7 had a documented urine pregnancy test on admission (47%). Of the 8 patients that had not had testing only 1 patient had documentation of contraceptive use prior to admission, the other 7 non-tested patients had no notes regarding their LMP/contraception. 2 patients who did not have a pregnancy test had in fact had a urinary drug screen on admission, this coincided with a time of approximately 1 month when there were no urine pregnancy test strips available on the ward. At this time serum bHCG or LMP were not routinely used. One of these patients was found one month later to be pregnant.

**Conclusion:**

We propose based on our findings that a more robust enquiry as to the risk of pregnancy should be conducted on admission for female acute inpatients. We have made recommendations that this should be in the form of a checklist to be conducted as part of the nursing admissions assessment such that if a urine sample is refused then a form detailing LMP, contraceptive use and any recent unprotected sexual activity will be completed. This can then be reviewed by the medical team prior to commencing medications. The use of this checklist will be reaudited between January-June 2022.

